# Procoagulant Phospholipids and Tissue Factor Activity in Cerebrospinal Fluid from Patients with Intracerebral Haemorrhage

**DOI:** 10.1155/2014/576750

**Published:** 2014-02-17

**Authors:** Patrick Van Dreden, Guy Hue, Jean-François Dreyfus, Barry Woodhams, Marc Vasse

**Affiliations:** ^1^Diagnostica Stago, 125 Avenue Louis Roche, 92635 Gennevilliers Cedex, France; ^2^Biochemistry Department, IBC, Rouen University Hospital, 76031 Rouen Cedex, France; ^3^Clinical Research Unit, Hôpital Foch, University of Versailles, 92151 Suresnes Cedex, France; ^4^HaemaCon Ltd, Bromley, Kent CT18 7TW, UK; ^5^Clinical Biology Department & EA 4531, Hospital Foch, 40 rue Worth, 92151 Suresnes Cedex, France

## Abstract

Brain contains large amounts of tissue factor, the major initiator of the coagulation cascade. Neuronal apoptosis after intracerebral haemorrhage (ICH) leads to the shedding of procoagulant phospholipids (PPLs). The aim of this study was to investigate the generation of PPL, tissue factor activity (TFa), and D-Dimer (D-Di) in the cerebrospinal fluid (CSF) at the acute phase of ICH in comparison with other brain diseases and to examine the relationship between these factors and the outcome of ICH. CSF was collected from 112 patients within 48 hours of hospital admission. Thirty-one patients with no neurological or biochemical abnormalities were used to establish reference range in the CSF (“controls”). Thirty had suffered an ICH, and 51 other neurological diagnoses [12: ventricular drainage following brain surgery, 13: viral meningitis, 15: bacterial meningitis, and 11 a neurodegenerative disease (NDD)]. PPL was measured using a factor Xa-based coagulation assay and TFa by one home test. PPL, D-Di, and TFa were significantly higher (*P* < 0.001) in the CSF of patients with ICH than in controls. TFa levels were significantly (*P* < 0.05) higher in ICH than in patients with meningitides or NDD. Higher levels (*P* < 0.05) of TFa were observed in patients with ICH who died than in survivors. TFa measurement in the CSF of patients with ICH could constitute a new prognostic marker.

## 1. Introduction

Intracerebral haemorrhage (ICH) is a type of stroke caused by bleeding within the brain tissue itself. Primary ICH accounts for ~80% of all incidences of ICH and is more likely to result in death or major disability than ischemic stroke or subarachnoid haemorrhage. ICH has a higher incidence among populations with a higher frequency of hypertension, including African Americans and Asian populations, possibly due to environmental factors (e.g., a diet rich in fish oils) and/or genetic factors [1]. In contrast to the declining incidence of ischaemic stroke in high-income countries, the incidence of ICH has been constant [2]. The 1-month fatality rate after ICH does not appear to have changed over the last few decades with rates of 25–35% in high-income countries and 30–48% in low-/middle-income countries [3].

Computed tomography (CT) is now widely available in the developed world and has become the diagnostic test of choice in ICH to determine the site of the haemorrhage and estimate the volume of the haematoma [4]. The ability of CT scans to detect ICH depends on the length of time between the bleed and the scan. However, up to 5% of subarachnoid haemorrhage (SAH) will have a negative CT scan.

Over the past decade, numerous studies have reported a positive correlation between S100B levels in blood or CSF and impaired neurological function. S100B is a calcium-binding protein concentrated in glial cells (although it has also been detected in definite extraneural cell types) and serum S100B is considered as a relevant diagnostic and prognostic tool in acute spontaneous ICH. However, a recent study in patients with ICH suggested that its sensitivity and specificity were not as high as previously described at least in patients with traumatic brain injury [5] and considerable evidence indicates that S100B is not a specific biomarker for brain damage [6].

During stroke, the blood-brain barrier (BBB) is compromised by endothelial cell death. Cytosolic contents released from injured brain tissues have the potential to cross the barrier. We hypothesise that the measurement of molecules which are expressed in high concentration in the brain but in trace amount in normal CSF could be used to monitor ICH severity and prognosis. As the brain is a major source of tissue factor (TF), the main coagulation cascade activator [7] and rich in phospholipids [8], we investigated the activity of procoagulant phospholipids (PPL) and the activity of tissue factor (TFa) in the CSF of patients with ICH. We compared the levels of these parameters in ICH and in other pathologies [bacterial or viral meningitis, patients with a ventricular drainage (VD) following brain surgery and patients with neurodegenerative diseases]. In addition, we examined the CSF levels of D-Dimer (D-Di) and tissue factor pathway inhibitor (TFPI) and analyzed the prognostic value of these parameters.

## 2. Patients and Methods

### 2.1. Patients

Fresh CSF was collected from 112 hospitalised patients by lumbar puncture in the first 2 days after admission (i.e., the early stage of events). Among these, 31 patients that had been admitted to the emergency ward with suspected meningitis or subarachnoid haemorrhage but were found to had no CSF biochemical abnormalities and normal neurological explorations were considered as controls. Of the remaining patients, 30 had suffered a cerebral haemorrhage, and 51 had other neurological disorders: 13 with confirmed viral meningitis, 15 with bacterial meningitis, and 11 with a neurodegenerative disease (NDD) Alzheimer's type; 12 were treated with ventricular drainage (VD) following brain surgery. ICH patients, defined as an acute and persisting focal neurological deficit, were to be admitted to the neurological department within six hours after symptom onset. No patients received intrathecal fibrinolytic therapy, antiplatelet agents, or prophylactic calcium channel blockers before sample collection. Informed consent was obtained from all appropriate family members.

The CSF was immediately placed on ice to prevent enzyme activation and transported to the laboratory where each was centrifuged at 1,000 ×g for 15 min to remove cells and debris. A small number of CSF samples were bloody and were additionally centrifuged for 15 minutes. Medical treatment was started soon after the lumber puncture when cranial CT had proved the existence of ICH.

### 2.2. Analytical Determinations

PPLs in the CSF were measured on a STA-R analyser using a factor Xa-based coagulation assay (STA Procoag-PPL—Diagnostica Stago, Asnières, France). The test consists of the measurement of clotting time in the presence of CSF, in a system in which a phospholipid-depleted substrate plasma makes the test dependent on the PPL of the test sample. Bovine factor Xa triggers the coagulation cascade downstream from factor Xa, thus eliminating the influence of coagulation factors acting upstream [9]. A shortening of clotting times is associated with increased levels of PPL activity. The effect of contamination with red blood cell (RBC) on the PPL determination was evaluated by adding a varying number of RBC to normal CSF after baseline PPL activity determination. The addition of RBC up to 55 × 10^9^/L in normal CSF did not change the time of PPL determination, while CSF specimens included in this study contain less than 45 × 10^9^ RBC/L.

TFa was measured by a one-stage kinetic chromogenic method [10]. This assay measures the ability of TF-FVIIa to activate factor X to factor Xa. The CSF samples were mixed with human factor VII and factor X and fibrin polymerisation inhibitor and incubated at 37°C allowing for the formation of TF/Factor VIIa complex. The secondary generation of factor Xa was measured by adding a specific factor Xa chromogenic substrate. Inter- and intra-assay coefficients of variation for this kit were 3.8% and 4.2%, respectively.

Free tissue factor pathway inhibitor (f-TFPI) was determined by ELISA (Asserachrom Free TFPI, Diagnostica Stago). D-Dimer (D-Di) was measured on a STA-R analyser by latex immunoassay (STA-Liatest D-Di, Diagnostica Stago) according to the manufacturer's instructions.

CSF proteins, glucose, chloride, and lactate measurements were done using the routine laboratory techniques (Cobas 6000, Roche Diagnostics, Meylan, France).

### 2.3. Statistics

All statistical calculations were done using NCSS, version 9 (NCSS LLC, Kaysville, UI, USA) and R statistical Package, version 3.0.2 (The R Statistical Foundation, Wien, Austria). Data are presented as median (1st and 3rd quartiles). Since all tests showed significant departure from normal distribution, between—group comparisons of continuous variables between different subgroups were performed using Kruskal—Wallis non parametric analysis of variance. When this led to global significance, pairwise comparisons were done between all groups and control and all groups and ICH, using Dunn's test. The Bonferronni-Simes correction was used to preserve the nominal significance level. Coefficients of correlation (*r*) were calculated using Spearman's rank test. A two-sided *P* value <0.05 was considered significant. For survivors to nonsurvivors comparison, a Mann-Whitney test was used. Receiver operating characteristic (ROC) analysis was performed to calculate cut-off values discriminating survivors and nonsurvivors.

## 3. Results

### 3.1. Variations of PPL, TFa, D-Di, and Routine Biochemical Parameters in the CSF of Patients and Controls

Clotting time in the PPL assay and levels of TFa and D-Di, in the CSF of the controls and patients are shown in Table 1. PPL (inversely related to clotting time), TFa, and D-Di were significantly higher in CSF from patients with ICH than in controls (*P* < 0.001). PPL was also significantly increased in comparison with controls in the different groups of patients analyzed, even in patients with NDD. PPL in patients with ICH was also significantly higher when compared to the different pathological groups, except in patients with VD and NDD.

When compared to controls, TFa was significantly increased only in patients with ICH. D-Di was significantly increased in patients with ICH and in patients with VD.

Compared to controls, CSF proteins, glucose, and lactates were significantly increased in patients with ICH. Significantly higher levels of CSF glucose were found in ICH patients when compared to patients with both types of meningitis and NDD and of CSF lactates when compared to NDD.

The levels of f-TFPI in the CSF of control and in patient CSF of the different groups were below the detection level (0.5 *μ*g/L, data not shown).

In the CSF of controls, a significant positive correlation (*P* < 0.001, *ρ* = 0.643) was found only between TFa and D-Di. In the CSF of patients with ICH, clotting time in the PPL assay was significantly inversely correlated (*P* = 0.005; *ρ* = − 0.53) with CSF lactate levels, which means that the quantity of PPL increases with the lactate level. In the CSF of patients with bacterial meningitis, a significant positive correlation (*P* = 0.02, *ρ* = 0.637) was found between TFa and D-Di and an inverse correlation between TFa and chloride (*P* = 0.02, *ρ* = − 0.614). In CSF of patients with VD, a positive correlation was found between glucose and lactate (*P* = 0.01, *ρ* = 0.761). 

### 3.2. Prognostic Values of PPL, TFa, and D-Di in the CSF of Patients with ICH

Significantly higher levels of TFa were observed in the CSF of patients with ICH who died compared with the survivors (*P* < 0.05). There was no significant difference in CSF D-Di levels (*P* = 0.48) and PPL (*P* = 0.07). ROC curve analysis indicated PPL to be more sensitive but less specific than TFa (Table 2).

## 4. Discussion

ICH has a mortality rate of 50%, and survivors may have significant neurological morbidity. Given the catastrophic consequences of missing the diagnosis and that both mortality and morbidity increase with delays in treatment, early diagnosis is essential and new approaches are needed to facilitate diagnosis. In addition to CT, identification of new biomarkers that denote the presence of ICH and contribute to an early diagnosis is welcome. They could have additional value if they can be used as a predictor of clinical outcome. Plasma S100B was previously shown to be increased in ICH and have a prognostic value [5, 11]. Increased levels of S100B in blood is however not specific for ICH, as increases occur in other neuropathologies including traumatic brain injury and extracranial malignancies and has been shown to represent ongoing neurogeneration [6]. Moreover, contradictory data interpretation exists with regard to the contribution of an altered blood-brain barrier to S100B serum levels [12].

The most frequent pathophysiologic mechanism of ICH seems to be a degenerative vessel wall change and consequently rupture of small penetrating arteries and arterioles. Recently it was shown that the plasma and the CSF of patients suffering from traumatic brain injury contain phosphatidylserine (PS) mainly of platelet and endothelial origin [13]. In this study, using a rapid assay [9] we found that PPL levels were significantly elevated in the CSF of patients with ICH in comparison with controls or other CNS pathologies and associated with a poor prognosis. The absence of correlation between PPL in the CSF and CSF proteins suggests that PPL appears in CSF, not because of changes in the BBB permeability but primarily because cells are damaged. This hypothesis is strengthened by the correlation between CSF lactate and PPL in this group of patients. In traumatic injury it has been shown that there is a difference in CSF phospholipid composition and the time of appearance of these phospholipids in patients with a poor outcome. In patients who died, phosphatidylserine (PS) and phosphatidylethanolamine were higher in the 48-hour time period following the traumatic injury [14]. Using a different methodology, based on the capture by annexin V of PS expressing microparticules, a similarly poor prognostic value associated with high levels of PS had been previously observed in patients with basal ganglia haemorrhage [15]. In our study, there was a trend for higher levels of PPL in the CSF of patients who died, but it did not reach a statistical significance. This can be due to a too weak number of patients in each group.

It was beyond the scope of this study, but it would, in future studies, be of interest to identify the origin of these cells in the different groups of patients tested in this study, since CSF PPL levels were significantly increased in each group, where different physiopathological mechanisms are suspected.

The brain is rich in TF. The cellular sources of TF are the astrocytes in the parenchyma, glia limitans, and arachnoid cells, with the latter two being in contact with the CSF [7, 16]. However little information is available on TF in the CSF of patients with diseases of the CNS in comparison with normal subjects. An increase in TF detected immunologically (TF : Ag) has been previously reported in patients after a subarachnoid haemorrhage [17, 18] and in patients with bacterial meningitis [19]. In this study, we used a functional TF assay. In normal CSF, TFa levels (0.73 pM/L) were higher than in plasma from healthy donors (0.25 pM/L) [10], which are similar to values obtained using an immunological assay (165 ± 139 pg/mL in plasma versus 868 ± 721 pg/mL in CSF) [20]. As observed by others [18], we could not detect f-TFPI in CSF from controls or from patients with subarachnoid haemorrhage. This TF-TFPI imbalance in normal and pathological CSF would tend to make it more procoagulant as the TFa cannot be neutralised. We also observed a significant increase in TFa in ICH patients, but in contrast with a previous study [19] not in patients with bacterial meningitis. This discrepancy between the two studies could be due to the proteolysis of TF by proteases secreted by bacteria, leading to the loss of TF activity, whereas the degraded/inactive TF is still recognised by antibodies used in the assay. TFa concentrations were higher in the CSF of patients with a poor outcome compared with those who survived. As TF is the major initiator of the coagulation cascade, it can be hypothesised that high CSF TFa levels are associated with generation of thrombin which is capable of releasing potent vasoconstrictors such as endothelin-1, serotonin, and platelet derived growth factor inducing a vasospasm [17]. In agreement with the hypothesis of an increased thrombin generation, elevated levels of prothrombin F1+2 and thrombin-antithrombin complexes were observed in the CSF of patients with ICH [17, 18]. It was suggested that elevation of CSF TF was predictive of vasospasm in subarachnoid haemorrhage and therefore associated with a poor prognosis [17]. However, this was not confirmed on a small series of patients [21] and has no prognostic value (independently of the appearance of a vasospasm). In our study, the prognostic value of the increase in TFa is higher than the increase in PPL, and it is clear that it deserves further investigation with a greater number of patients.

The third parameter studied was D-Di. In agreement with previous studies [22, 23], we observed increased D-Di levels in the CSF of patients with ICH compared with controls but did not identify any prognostic value for this parameter and were unable to find any previous studies in the literature evaluating the value of D-Di in CSF. Most studies investigated whether the presence of D-Di in the CSF can rapidly distinguish between a traumatic tap and ICH, but all with inconclusive results [22–24]. These discrepancies and the absence of prognostic value in our study can possibly be explained by the lack of specificity of CSF D-Di, since increased levels of D-Di were shown in the CSF of patients with different pathologies [24].

In conclusion, PPL and TFa assays presented in this study are rapid tests which can be performed in an emergency context of ICH and can provide useful diagnostic information. Further studies are required to better assess the relation between these markers and the occurrence of cerebral vasospasm or oedema formation and to evaluate whether the time-course of these factors may be helpful in predicting patient complications. The limitations of our study are the small sample size and relatively small number of patients included in each group. This makes it difficult to draw firm conclusions. These assays should be compared to new parameters such as copeptin or resistin which were recently described as having a prognostic value in ICH [25, 26].

## Figures and Tables

**Table 1:** Levels of procoagulant phospholipids (PPLs), tissue factor activity (TFa), D-Dimer (D-Di), and classical biochemical parameters in the cerebrospinal fluid of different groups of patients with central nervous system pathologies.

Variable (*P* ^§^)	Controls	ICH	Bacterial meningitis	Viral meningitis	VD	NDD
*n*	31	30	15	13	12	11
Clotting time for PPL assay (sec.) (*P* < 0.00001)	164 (158–174)*	125 (93–147)***	144 (136–165)^∗+^	151 (141–155)^∗+^	134 (132–147)***	142 (135–156)*
TFa (pM/L) (*P* < 0.00001)	0.74 (0.60–0.89)^*ε*^	2.36 (1.67–3.21)***	0.76 (0.68–0.83)^+ + +^	0.84 (0.74–0.92)^+ + +^	1.33 (1.12–1.60)	0.78 (0.62–0.89)^+ + +^
D-Di (mg/L) (*P* < 0.00001)	0.40 (0.17–0.58)^*β*^	0.82 (0.52–0.98)***	0.51 (0.43–0.59)^+^	0.44 (0.36–0.51)^*β*+ +^	0.54 (0.44–0.62)***	0.36 (0.23–0.52)^+ + +^
Protein (g/L) (*P* = 0.0006)	0.39 (0.24–0.48)	0.62 (0.44–0.85)^*β*∗∗∗^	0.58 (0.38–0.83)*	0.61 (0.55–0.88)***	0.28 (0.18–0.71)	0.45 (0.44–0.52)
Glucose (mmol/L) (*P* < 0.00001)	3.4 (3.2–3.9)	4.8 (4.1–5.4)***	3.1 (2.1–3.8)^+ + +^	2.8 (2.6–3.4)^+ + +^	4.0 (3.5–5.2)	3.6 (3.5–3.7)^+^
Chloride (mmol/L) (*P* = 0.57)	125 (124–128)	126 (120–132)	124 (117–132)	122 (120–132)	128 (125–132)	126 (123–128)
Lactate (mmol/L) (*P* < 0.00001)	1.67 (1.40–2.16)^*φ*^	2.93 (2.18–3.21)***	2.60 (2.10–3.76)^Γ∗^	2.77 (2.36–2.88)^*δ*∗^	2.66 (1.71 −3 .87)*	1.34 (1.30–1.44)^*α*+++^

Values are medians (1st and 3rd quartiles); *n* = number of patients enrolled.

^§^Global *P* for Kruskal-Wallis test *P < 0.05, ***P < 0.001 versus controls; ^+^ *P* < 0.05, ^++^ *P* < 0.01, ^+++^ *P* < 0.001 versus ICH Missing values: *α*: 5, *β*: 1, Γ:8, *δ*: 4, *ε*: 2, *φ*: 4.

ICH: intracerebral haemorrhage VD: ventricular drainage; NDD: neurodegenerative disease.

**Table 2:** Procoagulant phospholipids (PPLs), tissue factor activity (TFa) and D-Dimers (D-Di) in the CSF of survivor and nonsurvivor patients with ICH.

	Intracerebral haemorrhage					
	Survivors	Nonsurvivors	Cut-off	AUC	Sensitivity	Specificity
*n*	19	11				
TFa (pM) *P* = 0.016	1.85 (1.26–2.65)	3.21 (2.26–3.65)*	2.36	0.77	73.7	72.7
PPL (sec.) *P* = 0.07	143 (107–155)	100 (78–126)	106.6	0.70	78.9	63.6
D-Di (mg/L) *P* = 0.48	0.78 (0.51–0.98)	0.87 (0.62–1.23)	ND	ND	ND	ND

*P < 0.05, ICH survivors compared with ICH nonsurvivors, Mann-Whitney test.

Values are medians (1st and 3rd quartiles). *n* = number of patients.

AUC: area under the curve, ND: not determined.
